# The Diversity of Tone Languages and the Roles of Pitch Variation in Non-tone Languages: Considerations for Tone Perception Research

**DOI:** 10.3389/fpsyg.2019.00364

**Published:** 2019-02-26

**Authors:** Catherine T. Best

**Affiliations:** MARCS Institute, Western Sydney University, Penrith, NSW, Australia

**Keywords:** tone processing, non-native speech perception, prosodic hierarchy, tone language diversity, non-tone languages

All languages employ consonants and vowels as discrete contrastive subcomponents of the basic timing units of words (syllables). These two classes of phonemes are used to differentiate between words, whose meanings can be categorically changed by switching even a single vowel or consonant, as in <*pat*> vs. <*cat*> or <*pet*>. They populate the lowest level of the phonological hierarchy, the segmental tier, and both classes are obligatory across spoken languages. But only some languages also make use of *lexical tones*, contrastive sub-syllabic fundamental frequency (pitch) variations referred to as *tonemes* (e.g., Jones, 1944), which for those languages comprise a third class of phonemic elements. Perceptual researchers often assume tones to be suprasegmental (e.g., So and Best, 2010, 2011, 2014; Liu et al., 2018; Poltrock et al., 2018), i.e., to extend across the consonants and vowels of the target syllable. While in a phonetic sense tones extend across the voiced segments of a syllable, however, such observations may not straightforwardly reflect the more abstract phonological properties of tones (e.g., see Wang, 1967; Hyman, 2011a,b). Indeed, several tone phonologists claim that lexical tones function as segments in tone languages (e.g., Lin, 1989; Duanmu, 1990, 1994). For the following paragraphs we adopt that phonological view that lexical tones function in tone languages at the segmental level, along with consonants and vowels. However, we return later to consider their phonological status and its relevance for understanding lexical tone perception by native and non-native listeners.

Unlike consonants and vowels, lexical tones are optional^1^. Many languages of Europe, the Americas, Oceania, Africa and even Asia function perfectly well without them. But lexical tones are, nevertheless, a popular option. They are employed in 60–70% of existing languages (Yip, 2002), including many Asian, African and indigenous American languages as well as a few European and South Pacific languages (Maddieson, 2013). It is important to note, nonetheless, that lexical tone forms and usage vary widely across tone languages (e.g., Hyman, 2011a, 2016; Remijsen, 2016)^2^. Some include tonemes with temporally-changing pitch trajectories (contour tone languages) while others use only level pitches (register tone languages). Some rely only on pitch specifications for tone contrasts while others have been claimed to also incorporate phonation distinctions^3^. Some have seven or more contrastive tones while others have as few as two. Some apply tone values to all syllables while others restrict tones to accented syllables of specific words (lexical pitch accent^4^). Some use tones only for stem morphemes while others use tone to mark grammatical or morphological alternations. Tone languages also differ in their degree of reliance on lexical tone distinctions, ranging from extensive, i.e., high functional load, to quite restricted use, i.e., low functional load.

Moreover, languages that lack lexical tones (non-tone languages) are far from devoid of systematic pitch variations. All spoken languages use pitch and contour paralinguistically, e.g., to convey information about emotions and talker gender and age. More importantly for our discussion of lexical tones, all languages also use pitch variation linguistically to mark intonation distinctions at supra-syllabic (metrical) levels of the phonological hierarchy: prosodic word, phonological phrase, intonational phrase, and utterance tiers (the prosodic hierarchy: e.g., Beckman and Pierrehumbert, 1986; Nespor and Vogel, 1986; Selkirk, 1986; Pierrehumbert and Beckman, 1988), which are most often examined using the ToBI (Tones and Break Indices) framework and transcription system (see Beckman et al., 2006), an approach that has also been applied to lexical tones (e.g., Francis et al., 2008). Clearly, then, phonological use of pitch distinctions is familiar to non-tone language speakers, at higher metrical levels of their language.

The crucial difference between tone and non-tone languages is that tone languages use contrastive pitch specifications at every level of the phonological hierarchy, whereas non-tone languages have a gap in contrastive use of pitch at the segmental level. As a result, non-tone language speakers are likely to perceive non-native lexical tones in terms of paralinguistic information and/or as native-language (L1) prosodic distinctions. For example, they may perceive non-native lexical tones as L1 intonational phrase (e.g., Hallé et al., 2004) and/or stress contrasts (e.g., So and Best, 2010, 2011, 2014). Such a discrepancy in phonological tiers between the lexical tones of the non-native stimulus language and the higher prosodic level(s) at which non-tone L1 listeners perceive the pitch variations as distinctive may explain why non-tone L1 adults often err in perceiving, producing and remembering the lexical tones of names and words in a tone language (McGinnis, 1997), including even very proficient English-L1 speakers of L2 Mandarin (Wong and Perrachione, 2007). In tone word training studies, non-tone L1 listeners learn novel words' consonant-vowel patterns faster and more accurately than their lexical tones (Wong and Perrachione, 2007). They also display substantial individual variation in learning, which correlates with variations in their tone discrimination performance in non-lexical tasks (e.g., Wong et al., 2007; Chandrasekaran et al., 2010). Nonetheless, learning tones in words is more challenging than mere tone discrimination, which is clearly above chance even prior to training (e.g., 78% correct discrimination in a pre-test: Wong and Perrachione, 2007)^5^.

Unique insights into how language experience shapes phonological knowledge could be gained from studies of non-native and native tone perception that exploit the diversity of lexical tone systems, and probe how a range of contrast types are perceived in relation to prosodic distinctions at higher tiers of the phonological hierarchy. Most prior studies of lexical tone perception by infants and young children, however, have drawn their target stimuli and native listeners from a small set of Asian languages that have contour tone systems, though there are some exceptions (e.g., Yoruba, an African register tone language: Harrison, 2000; Japanese, an Asian pitch accent language: Nazzi et al., 1998; Sato et al., 2009; Ota et al., 2018). The non-native listeners have often been non-tonal L1 speakers naïve to the target tone language, though in a few studies their L1s have been pitch accent languages (e.g., So and Best, 2010) or other contour tone languages (e.g., So and Best, 2010, 2011, 2014; Reid et al., 2015). Another potential limitation of much prior research with young children is that often only discrimination has been tested (e.g., Harrison, 2000; Mattock and Burnham, 2006; Mattock et al., 2008; Yeung et al., 2013; Liu and Kager, 2014; Hay et al., 2015; Cheng and Lee, 2018). However, more recent studies have extended the investigation to word recognition and learning (Singh and Foong, 2012; Singh et al., 2014; Hay et al., 2015), including a number of papers in this Special Topic volume (e.g., Liu and Kager, 2018; Ota et al., 2018; Burnham et al., 2019; and several other papers discussed below). Other recent advances include studies on the developmental relationship between perception of lexical tones and perception of higher-tier linguistic information such as stress and prosody (Quam and Swingley, 2010; Liu and Kager, 2014; Singh and Chee, 2016; Choi et al., 2017; Ma et al., 2017) and paralinguistic features such as pitch variations that convey emotions (e.g., Kager, 2018).

The six articles I was invited to comment on have each extended that recent progress in our understanding of the early development of native and non-native perception of lexical tones. All expand beyond the issues addressed in most previous research, although five of them maintain the typical focus on Asian contour tone languages, specifically the most-often-studied language, Mandarin, and a second widely-spoken Chinese language, Cantonese. Chen et al. (2017) found that infants learning Dutch, a non-tone language, discriminated both a difficult Mandarin contour tone contrast (T2-T3) and matched tritone piano melodies at 12 but not 4 months, despite lacking exposure to lexical tones in their environment. The authors interpret these results as evidence that development of pitch contour perception is mediated by domain-general rather than language-tuned mechanisms. In a second paper, however, although both Mandarin-learning and English-learning infants also discriminated another Mandarin tone contrast (T1-T3) better at 12 than at 6 months, the Mandarin infants showed significantly greater improvement, which indicates that language-specific experience does enhance lexical tone discrimination (Tsao, 2017). Moreover, in a different categorial discrimination task both 4- and 13-month-old Mandarin-learning infants discriminated the Mandarin T2-T3 contrast (same as in Chen et al., 2017), but Mandarin 2-year-olds *failed* to detect T2-T3 tone mispronunciations of known words (Shi et al., 2017). The latter finding mirrors a previously-observed discrepancy between infants' basic discrimination of a consonant contrast as compared to their later poor recognition of that same contrast when it occurs in words (Stager and Werker, 1997).

Older children were the participants in the other three articles, two of which examined Cantonese-learning children. In one, 3-year-olds failed to perceive or produce Cantonese tones like adults but, consistent with a classic speech development hypothesis they were more accurate in tone perception than production (Wong et al., 2017). In the other, Cantonese 3rd-graders' lexical tone sensitivity was found to correlate with their sensitivity to lexical stress in L2-English words (Choi et al., 2017). The remaining article (Ramachers et al., 2017) took an important additional step away from the past by using a European pitch accent language, Limburgian, rather than an Asian contour tone language in which tones carry high functional load in the lexicon but no grammatical function. Limburgian's binary level-tone distinction, which is embedded in a complex intonation system, carries a low functional load, but contributes both to lexical items and to a morphological alternation for a few frequent nouns in which falling pitch indicates plurality. No evidence of effects of language experience was found for Limburgian- versus Dutch-learning 2.5- and 4-year-olds' learning of novel Limburgian words with lexical tone: children of both ages were sensitive to tone mispronunciations of the newly-learned words. The authors inferred that the children's lexical representations for the novel items included tone specifications.

This set of papers individually and together advance our knowledge about the development of young children's perception and production of lexical tones, of their phonological representation of tones in words, and of the impact that speaking a native tone language may have on children's perception of lexical stress in a non-tone second language they are learning. Nonetheless, there is still a long way to go in understanding the role of experience in perception and phonological representation of lexical tone contrasts. Ideally, future research should include a wider range of non-Asian languages, including register tone as well as contour tone languages, and wider variations in the functional loads and morpho-grammatical functions of lexical tones across languages. Cross-language comparisons across a wider range of lexical tone systems will be needed to identify where, how and why perceptual assimilation of non-native lexical tones to higher prosodic tiers in the native languages of non-tone L1 listeners may break down. Similarly, use of the full range of lexical tone types and systems will be needed to determine whether, when and how young non-tone language learners may shift from perceiving non-native lexical tones as potential segmental contrasts (like consonants and vowels) to assimilating them as native prosodic patterns, and on the other hand to better understand how and when young learners of tone languages begin to tease apart lexical tones (segmental tier) from not only paralinguistic indexical information (talker identity, gender, emotion etc.) but also linguistic prosodic information in their language.

Understanding the phonological status of lexical tones could provide an important linguistic basis for predicting and interpreting both native and non-native tone perception and early learning. However, it has not yet been resolved whether the lexical tones of tone languages serve suprasegmental or segmental functions, and in the latter case whether they constitute a third class of phonological segments or serve as phonological features of vowels or of consonants. As briefly summarized in the following paragraphs, certain sources of evidence and/or theoretical analyses appear to be consistent with each of these possibilities. Unfortunately, the nature of the evidence differs among them, making it difficult to decide among them. Further research and theoretical analyses will be needed to tease them apart. It is likely that the answer will depend on whether the approach focuses on tone production and phonological processes in lexical tone languages, or whether the approach focuses instead on native or non-native perception. With the former approach the answer may vary depending on what types of tone systems the target languages have, whereas with the latter approach the answer should vary according to whether the listener groups have tone or non-tone L1s.

The question of the suprasegmental vs. segmental status of lexical tones in tone languages has been addressed primarily via phonological analysis of diachronic and synchronic data on tones as produced in a range of languages. In classic generative phonology tones were considered to be segmental in nature (e.g., Chomsky and Halle, 1968). Furthermore, as noted earlier, Duanmu (1990, 1994) and Lin (1989) also concluded from the phonological evidence that tones function as segments in tone languages, and of course for their native speakers. Based on cross-language phonological analyses, Hyman also concluded that tones serve segmental functions in tone languages, though he reasoned that in addition, unlike consonants and vowels, tones also can and do serve metrical (suprasegmental) functions. Thus, the concensus from a phonological point of view is that lexical tones function as segments in the languages that employ them contrastively, although they can also serve suprasegmental functions in those languages.

This leads us to the next question: do lexical tones constitute a third class of phonological segments in addition to consonants and vowels in tone languages, or do they instead serve as optional phonological features of vowels or consonants? In the classic generative phonology framework of (Chomsky and Halle, 1968), lexical tones were treated as an optional set of vowel features, i.e., *not* as a separate third class of segments. On the other hand, several lines of phonological evidence suggest that lexical tones may function as consonantal features (rather than as a third segmental class) in tone languages. Firstly, the emergence of lexical tones during the historical evolution of a language (tonogenesis) is much more likely to arise via diachronic changes in laryngeal features of consonants, e.g., through trans-phonologization of voicing contrasts, than from diachronic changes in vowels (see Maddieson, 1984; Whalen et al., 1993; Ratliff, 2015; Remijsen, 2016; for ongoing consonant voicing-related tonogenesis in Seoul Korean, see Silva, 2006a,b). Secondly, some articulatory studies of speech production in tone languages have demonstrated that the laryngeal gesture that produces a lexical tone is coupled with the constriction gesture for the onset consonant of the tone-bearing syllable rather than being coupled with its vowel nucleus (Gao, 2009; Mücke et al., 2012; Hu, 2016). However, a recent articulatory study instead found that certain Mandarin tones differentially shift tongue body position in production of adjacent vowels (Shaw et al., 2016), which may be consistent with viewing them as vowel features. Alternatively, the phonological analyses of Duanmu (1990, 1994), Lin (1989) and Hyman (2011a,b) posit that although tones interact with consonants and vowels in various ways, depending on the specific tone language, tones are autonomous. This implies that in their views, tones are a separate, optional third segmental class, distinct from vowels and consonants. Thus, there does not appear to be a clear consensus from phonological and articulatory studies as to whether lexical tones function as a third, separate class of segments, or instead serve as vowel features or consonant features. Nor do neurocognitive studies resolve the issue. Some report a dissociation of tone processing from both consonant and vowel processing (Li et al., 2010), while others report partial dissociation of brain activation during tone vs. vowel production (Liu et al., 2006), and still others observed similar production difficulties with tones and consonants, but not with vowels, in non-fluent aphasic speakers of Mandarin (Packard, 1986).

Can we form a clearer picture based on existing cross-language tone perception studies? On the one hand, many reports on early developmental changes in non-native lexical tone perception appear compatible with the idea that tones are phonologically associated with consonants. For example, English-learning infants have been found to discriminate non-native Mandarin tone contrasts at 6 months but not at 9 months (e.g., Mattock and Burnham, 2006), consistent with numerous reports of a developmental decline around 10 months in discrimination of many non-native consonant contrasts and at odds with reports of an earlier decline at 5–6 months for non-native vowel contrasts (e.g., Werker and Tees, 1999). On the other hand, findings from a recent eye-tracking study of novel tone-language word learning by native, non-native tone L1 and non-native non-tone L1 adults indicate that tone processing appears to be more tightly time-locked to the vowel than the consonant onset in the words (Poltrock et al., 2018).

Further complicating things are other developmental findings suggesting that language-specific changes in consonant perception appear somewhat earlier, by 8 months, in French-learning than English-learning infants (Hoonhorst et al., 2009). And language-specific differences may emerge even earlier, by 4 months, in non-native English- and Mandarin-learning, and native Cantonese-learning infants' perceptual preferences for Cantonese tones (Yeung et al., 2013), in contrast to the previously reported language-specific decline in discrimination of non-native tones by 9 months (Mattock and Burnham, 2006). Yet other studies indicate instead that even 2- to 3-year-old monolingual tone language learners are not yet adultlike in their learning and recognition of spoken words, for which they are more strongly affected by vowel variation than tone variation (Ma et al., 2017), and they may not be able to perceptually disentangle the intonational vs. lexical basis for pitch variations until 4–5 years of age (Singh and Chee, 2016). In another study of monolingual Mandarin learners, however, 2- to 3-year-olds showed greater sensitivity to lexical tone mispronunciations than vowel or consonant mispronunciations of just-learned novel Mandarin words, whereas 4- to 5-year-olds reversed that pattern, showing greater sensitivity to vowel or consonant mispronunciations than to tone mispronunciations (Singh et al., 2015). By comparison, in a study of monolingual English- and monolingual Mandarin-learning children both groups detected either tone or vowel mispronunciations of just-learned novel Mandarin words at 18 months, but only Mandarin-learning children detected the tone mispronunciations at 24 months (Singh et al., 2014). In sum, then, existing perceptual investigations also fail to provide a clear answer to the question of whether tones form a separate segmental class or instead serve as features of vowels or consonants.

The challenge for further research is how to design tests of whether young children, or adults for that matter, perceive tones as features of consonants or vowels or as different from both, and of how that pattern may differ for native listeners vs. non-native listeners/learners of different types of tone languages or non-tone languages. Future research will also need to take into account that all languages, whether or not they use lexical tones, employ prosodic pitch distinctions at higher tiers of the phonological hierarchy. This means that speakers of so-called non-tone languages are not lacking entirely in experience with phonological information being conveyed by pitch variations, and can refer to native pitch settings at a higher tier of the prosodic hierarchy when perceiving non-native lexical tones. Conversely, it also means that for speakers or learners of a tone language there is potential for ambiguity or confusion over which phonological tier is being represented by a given tonal pattern. Such confusions could be the root cause of apparent developmental “dips” in tone sensitivity even in children whose native language uses lexical tones.

A key unanswered question for listeners from non-tone L1s is whether and how assimilating tones to native prosodic contrasts may help or hinder learning the lexical tones of words in a tone language. More specifically, it is an open question whether and how cross-tier perceptual influences differ quantitatively and/or qualitatively from perceiving non-native consonant and vowel contrasts with reference to same-tier native contrasts (for an excellent step toward addressing this see Braun and Johnson, 2011). These issues need to be carefully considered in any attempt to extend existing theoretical models of non-native and L2 speech perception, such as the Perceptual Assimilation Model (PAM: Best, 1995; Best and Tyler, 2007) or the Speech Learning Model (SLM: e.g., Flege, 1995), to the perception of non-native lexical tones by non-tone L1 listeners. Both models were developed specifically to account for cross-language perception of non-native consonants and vowels with reference to native segments, and can be extended fairly straightforwardly to predicting discrimination and categorization of non-native tones by adult listeners whose L1s are other tone languages, i.e., within the segmental tier. But neither model was designed to address the cross-tier perceptual relationships that are likely to come into play in non-native tone perception by listeners of non-tone L1s. Nonetheless, some studies have begun to examine perceptual assimilation of non-native tones to native intonation distinctions in non-tone listeners (e.g., So and Best, 2010, 2011, 2014) and the results suggest that such assimilations may be less categorical than are assimilations to another lexical tone system. The most comprehensive understanding of native and non-native tone perception and its development is likely to require studies in which the target stimuli are taken from a wider range of types of tone languages, and the listeners' L1s are representative of a wider range of tone and non-tone languages. There is still much to learn about perception of lexical tones, and how it changes developmentally in both native and non-native listeners.

## Author Contributions

The author confirms being the sole contributor of this work and has approved it for publication.

### Conflict of Interest Statement

The author declares that the research was conducted in the absence of any commercial or financial relationships that could be construed as a potential conflict of interest.

## Footnotes

^1^While lexical stress and gemination are also optional (non-obligatory) phonological features used for lexical contrast, they both are defined across multiple timing units. Lexical stress is a contrastive relationship realized across two or more syllables, while gemination involves repetition of the same segment across two adjacent morae, either within a syllable or across a syllable/morpheme boundary. Given our focus on lexical tones, we will not discuss them except if/as relevant to perception of tones.

^2^In addition, neither phonetic nor phonological notation for tones has been standardized or widely adopted to the same extent as for consonants and vowels (International Phonetic Alphabet [IPA], 2015). There are a number of competing and inconsistently used systems. Chao (1930) numbers (“letters”) have been adopted most often, primarily but not only for Asian languages. However, even when used, Chao numbers are applied within each language relativistically, making direct comparison between tones of different languages not as straightforward as one might expect. The IPA offers a schizoid choice between tone diacritics on the vowel or pictographic symbols placed next to the syllable; neither are used as widely as Chao numbers. And some researchers instead use idiosyncratic, language-specific tone symbols (e.g., Thai) and/or names that are sometimes but not always English-lexified (e.g., Mandarin *rising, falling, dipping, high level*; but Vietnamese *s*$\overset{´}{ă}$*c, ngang, ngà, huy*$\overset{`}{ê}$*n, h*ô*i* and *n*$\underset{{}^{\circ}}{ǎ}$*ng* [or merged *hôi*-*na*$\underset{{}^{\circ}}{â}$*ng* in South Vietnamese]). None of these notation approaches systematically reflects effects of phonetic context and sandhi rules on the phonetic form of tones as they are actually realized in connected speech.

^3^As these claims have referred to creaky voice (very widely spaced pitch pulses) and glottalization (temporary lack of pitch pulsing) it is not entirely clear to me that they are necessarily categorically different from pitch specification. For example, perhaps they could indicate *very* to *maximally low* pitch.

^4^While it remains a matter of debate whether lexical pitch accent is a type of lexical tone, for heuristic purposes, languages that use only pitch accents, such as Japanese, are considered tone languages in this paper. They are assumed to be specified at the segmental tier of the phonological hierarchy in such languages, rather than at the higher timing tiers, as Duanmu and Lin have posited for non-pitch-accent tone languages.

^5^Similar findings have been reported for discrimination vs. higher-level perceptual tasks involving non-native lexical stress contrasts (e.g., Skoruppa et al., 2009, 2013)
